# Imunocapture Magnetic Beads Enhanced and Ultrasensitive CRISPR-Cas13a-Assisted Electrochemical Biosensor for Rapid Detection of SARS-CoV-2

**DOI:** 10.3390/bios13060597

**Published:** 2023-05-31

**Authors:** Yao Han, Fan Li, Lan Yang, Xudong Guo, Xue Dong, Mengwei Niu, Yaxuan Jiang, Lin Li, Hao Li, Yansong Sun

**Affiliations:** 1 State Key Laboratory of Pathogen and Biosecurity, Beijing Institute of Microbiology and Epidemiology, Beijing 100071, China; hanyaohyhy@163.com (Y.H.); lihao1@bmi.ac.cn (H.L.); 2 Chinese PLA Center for Disease Control and Prevention, Beijing 102206, China; 3 College of Public Health, Zhengzhou University, Zhengzhou City 450001, China

**Keywords:** bioanalytical chemistry, biosensor, CRISPR Cas13a, electrochemistry, trans-acting cleavage

## Abstract

The rapid and ongoing spread of the coronavirus disease (COVID-19), caused by severe acute respiratory syndrome coronavirus 2 (SARS-CoV-2), emphasizes the urgent need for an easy and sensitive virus detection method. Here, we describe an immunocapture magnetic bead-enhanced electrochemical biosensor for ultrasensitive SARS-CoV-2 detection based on clustered regularly interspaced short palindromic repeats (CRISPR) and CRISPR-associated (Cas) proteins, collectively known as CRISPR-Cas13a technology. At the core of the detection process, low-cast and immobilization-free commercial screen-printed carbon electrodes are used to measure the electrochemical signal, while streptavidin-coated immunocapture magnetic beads are used to reduce the background noise signal and enhance detection ability by separating the excessive report RNA, and a combination of isothermal amplification methods in the CRISPR-Cas13a system is used for nucleic acid detection. The results showed that the sensitivity of the biosensor increased by two orders of magnitude when the magnetic beads were used. The proposed biosensor required approximately 1 h of overall processing time and demonstrated an ultrasensitive ability to detect SARS-CoV-2, which could be as low as 1.66 aM. Furthermore, owing to the programmability of the CRISPR-Cas13a system, the biosensor can be flexibly applied to other viruses, providing a new approach for powerful clinical diagnostics.

## 1. Introduction

Rapid and accurate point-of-care testing (POCT) for nucleic acid detection is crucial for emergency disease confirmation and treatment and epidemic prevention and control [1]. Currently, the most widely used method for nucleic acid detection is polymerase chain reaction (PCR), owing to its high sensitivity and specificity. However, PCR analysis requires well-equipped laboratories, large and expensive equipment, a professional tester, and long turnover times, which limits its ability to be used in POCT [2,3]. Finding a simple, rapid, and accurate detection system, which can satisfy the needs of a variety of application scenarios, has therefore become a popular trend in the fields of clinical diagnosis and pathogen detection.

Recently, there have been significant developments in using clustered regularly interspaced short palindromic repeats (CRISPR) and CRISPR-associated (Cas) proteins (e.g., Cas9, Cas12a, Cas13a) in the detection of nucleic acids by exploiting the trans-cleavage activity of the CRISPR-Cas system, which can indiscriminately cleave surrounding reporter RNA/DNA (near and around the target RNA/DNA). Together with isothermal amplification technology (e.g., rolling circle amplification; loop-mediated isothermal amplification; recombinase-aided amplification), easy read-out methods, and a small heater, all CRISPR detection steps can be easily performed in any setting, demonstrating great potential for POCT. Dai et al. [4] first reported on a promising electrochemical sensing CRISPR system that could detect DNA and proteins based on the CRISPR-Cas12a system. This system uses a nonspecific single-stranded (ss) DNA reporter, designed with a methylene blue (MB) electrochemical tag, and provides a new cost-effective and portable biosensing strategy for detecting nucleic acids. This new strategy has the advantages of both CRISPR technology, which includes high sensitivity and specificity and the ability to be programmed, and an electrical biosensor. Such CRISPR-based systems are simple, low-cost, and are emerging as promising tools for sequence-specific nucleic acid detection [2,4,5,6,7,8,9,10,11,12,13,14,15,16,17,18,19,20,21].

Researchers have improved the detection performance of the electrochemistry CRISPR platform for pathogenic or cancer-related nucleic acids, achieving ultrasensitive and accurate results. However, most of these biosensors require the electrochemical probes to be immobilized on the electrode surface [4,5,6,7,8,11,12,13,14,15,17,18,19,20], which is hard to preserve and requires preparation a few days before use [8]. Furthermore, the immobilization process is complicated and time-consuming. It may result in a steric hindrance effect leading to a reduction in cleavage efficiency and selectivity on the heterogeneous surface [22], thereby limiting the use of the biosensors in POCT. Certain other electrochemical CRISPR biosensors use specially fabricated electrodes, such as electrodes modified with graphene oxide [16] or gold nanoflower nanocomposite [14], to enhance the amount of probe captured by the electrode and thus the electrochemical signal. Electrodes optimization, such as TiO_2_ [23] and Carbon Nanofiber–Gold Nanocomposite [24] modification, or solution system compound optimization, such as concentration of Mg^2+^ and a related protein, to enhance the electrochemical ability is a good option. However, these types of electrodes require elaborate optimization of the manufacturing process, and the cost and complexity of the fabrication process are not competitive owing to the repeatability of the analysis and POCT requirements. Finally, the remainder of the electrochemical CRISPR biosensors which have been developed exhibit limited sensitivity [2,13,21], which prevents their use in POCT.

In view of the above, we aimed to develop an immobilization-free, easy-to-fabricate, simple electrode that could be used as an electrochemical CRISPR biosensor, overcoming the indicated limitations. In doing so, we exploited the properties of magnetic beads. The development and application of magnetic beads in separation and detection methodologies have attracted considerable interest in recent years. This is mainly because of their chemical and physical stability, high biocompatibility, high surface area, low toxicity, and versatility [25]. The size of the magnetic beads can be tuned as needed, ranging from nanometers to a few millimeters, and the magnetic beads with their linked molecules can be rapidly aggregated and separated from the solution. Thus, immunocapture material-coated magnetic beads, such as streptavidin (SA)-coated beads, can be used for separation of the excessive DNA/RNA electrochemical probes to decrease the electrochemical signal of a negative control group, which leads to better electrochemical signal performance.

Here, we describe the development and testing of an immobilization-free, reverse transcription recombinase-aided amplification (RT-RAA)-based CRISPR/Cas13a electrochemical biosensor that employs SA-coated magnetic beads for the detection of SARS-CoV-2.

## 2. Materials and Methods

### 2.1. Materials

In this study, a biotin-RNA-MB reporter was synthesized and purified by Takara (Dalian, China). Other oligonucleotides, including the primer sets, crRNA, and RNA-FQ reporter (fluorescent reporter ssRNA labeled 6-FAM on the 5′ side and BHQ-1 on the 3′ side), were synthesized by Beijing Tianyi Huiyuan Bioscience and Technology Inc. (Beijing, China) (Table S1). Screen-printed carbon electrodes were purchased from Red Matrix China Co., Ltd. (Guangzhou, China). Dynabeads^TM^ M-280 streptavidin was purchased from Invitrogen (Carlsbad, CA, USA). LwaCas13a protein (Cas13a protein expressed by Leptotrichia wadei) was purchased from GenScript (Nanjing, China). Magnesium chloride (MgCl_2_), sodium chloride (NaCl), Tris-hydrochloride (Tris-HCl), chloroform (CHCl_3_), and Ethylene Diamine Tetraacetic Acid (EDTA) were purchased from Sinopharm Chemical Reagent Co., Ltd., Shanghai, China, and used without further processing. MB was obtained from Sigma-Aldrich. HEPES buffer solution was purchased from GIBCO (Invitrogen). A COVID-19 Nucleic Acid Detection Kit (CRISPR flow immunochromatographic method) was purchased from Zhongtest Biotechnology Co., Ltd. (Hangzhou, China). A COVID-19 Nucleic Acid Detection Kit (fluorescence PCR method) was purchased from Da and Gene Co., Ltd. (Guangzhou, China). The ribonucleotide solution mix and T7 RNA polymerase were purchased from New England Biolabs LTD (Beijing). Novel Coronavirus Nucleic Acid Standard (High Concentration) was purchased from the Research and Management Center of Standard Substances, Chinese Academy of Metrology.

### 2.2. RT-RAA Amplification

For target amplification, the RAA reaction was performed using a Novel Coronavirus 2019-nCoV Nucleic Acid Test Kit (CRISPR Immunoassay) following the manufacturer’s instructions (product no. 211104006), which included freeze-drying the primer sets and other necessary substances in a reaction tube. Briefly, lyophilized reagent pellets containing 0.4 μM of forward and reverse primers in the kit were suspended in 17.5 μL of the supplied rehydration buffer, 30 μL of target RNA template, and 2.5 μL of magnesium acetate. The reaction mixture was then subjected to amplification at 42 °C for 30 min.

### 2.3. CRISPR/Cas13a Fluorescence Assay

CrRNA/Cas13a collateral cleavage was carried out, as previously reported in [26], with slight modifications. The working concentrations of the components used in the fluorescence CRISPR activation assay were as follows: 20 mM HEPES, 2 mM ribonucleoside triphosphates (rNTP), 40 nM LwCas13a, 120 nM crRNA, 200 nM RNA-FQ reporter, 10 mM MgCl_2_, 4 IU/μL RNase inhibitor murine, 2 IU/μL T7 RNA polymerase, 5 μL nucleic acid amplification products, and the total reaction volume was 25 μL.

For real-time fluorescence monitoring, the reactions were incubated for 60 min at 37 °C in a LightCycler^®^ 96 System (Roche, Switzerland). The fluorescence of the trans-cleavage reaction was measured every 2 min with excitation at 495 nm and emission at 520 nm.

### 2.4. CRISPR/Cas13a Electrochemical Assay

For the electrochemical analysis of trans-cleaved reporters, the cleavage assay was performed using previously described fluorescence CRISPR methods [27], with slight modifications. The working concentrations of the components used in the electrochemical CRISPR activation assay were as follows: 25 mM HEPES, 2.5 mM rNTP, 75 nM Cas13a, 50 nM crRNA, 1 μM biotin-RNA-MB reporter, 25 mM MgCl_2_, 1 IU/μL T7 RNA polymerase, 2 μL nucleic acid amplification products, and RNase-free water to a final volume of 40 µL. The reactions were incubated for 30 min at 37 °C.

Electrochemical measurements were performed using a CHI 660E electrochemical workstation (CH Instruments Co., Shanghai, China). For SWV tests, 40 µL of the supernatant was added dropwise to the screen-printed electrode surface and scanned by electrochemical methods. SWV was performed at 50 Hz with an amplitude of 50 mV and a potential increment of 4 mV in the range of −0.5 to 0 V. The supernatant was tested using the electrochemical method after being dropped onto the screen-printed electrode for about 1 min. Finally, we observed the current value (peak current value after subtracting the background) from SWV and analyzed the data.

A cutoff method was used to judge the detection result. The cutoff current value was defined by the average current value plus three times the standard deviation (SD) of the negative control (NC) group for determining positivity and negativity [28].

### 2.5. Immunocapture Separation

First, after mixing thoroughly, 30 μL of commercial SA-coated magnetic bead turbidity in a tube was taken and placed on a magnetic stand for 1 min; the supernatant was discarded. Resuspended SA-coated magnetic beads were then added in 40 μL of ddH_2_O, the tube was placed on a magnetic stand for 1 min again, and then the supernatant was discarded. The electrochemical CRISPR reaction mixture was added to the SA-coated magnetic beads and mixed. Finally, the tube was placed on a magnetic stand for 1 min before the supernatant was collected for square wave voltammetry (SWV).

### 2.6. RT-qPCR Assay

For comparison of the detection limit and clinical sample testing of the different methods, Reverse transcription quantitative real-time PCR (RT-qPCR) was performed using a commercial RT-qPCR kit (Da An Gene Co., Ltd., Sun Yat-sen University, Guangzhou, China). Although this kit can detect both SARS-CoV-2 ORF1ab and N genes, the sensitivity analysis focused on the N gene. The RT-qPCR reaction system and PCR conditions were as described in the kit instructions. A cycle threshold (Ct) value ≤40 was defined as a positive result, whereas a Ct value >40 was defined as negative.

### 2.7. Statistical Analysis

A Student’s *t*-test or Welch’s *t*-test was performed to compare different groups, and all data were presented as the mean ± SD of at least three independent experiments.

## 3. Results and Discussion

### 3.1. Design of the Electrochemical Biosensor

**Figure 1:**
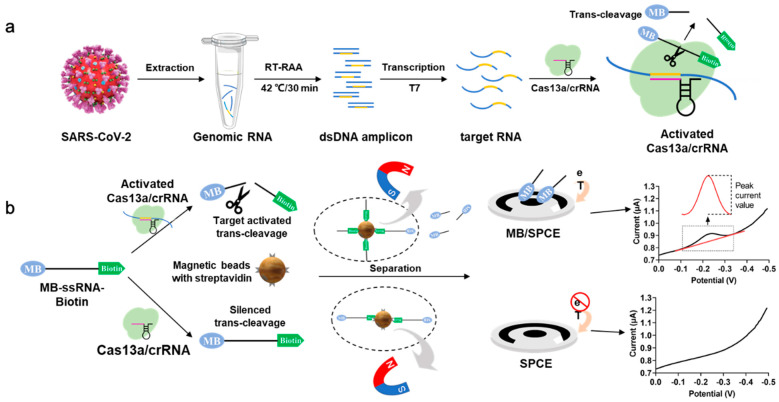
Principle of the electrochemical Clustered Regularly Interspaced Short Palindromic Repeats (CRISPR) biosensor for detecting severe acute respiratory syndrome coronavirus 2 (SARS-CoV-2) RNA. (**a**) SARS-CoV-2 RNA is extracted from a sample, reverse transcription recombinase-aided amplification (RT-RAA)-amplified, T7 transcribed, and then subjected to the LwaCas13a (Cas13a) trans-cleavage reaction. The Cas13a-crRNA duplex binds target SARS-CoV-2 RNA (140 nt), which activates the trans-cleavage activity. The reRNA, modified with biotin and methylene blue (MB), is then cleaved, and the unbound MB-oligonucleotide is used in the electrochemical measurement. (**b**) The main process of the electrochemical CRISPR biosensor. When the target RNA is present, the function of Cas13a-crRNA is activated, and the reRNA is cleaved into MB-oligonucleotide and biotin-oligonucleotide. The extra uncleaved reRNA and biotin-oligonucleotide are removed by SA-coated magnetic beads, leaving the MB-oligonucleotide in the solution, which could be detected by electrochemical measurement. When the target RNA is absent, the function of Cas13a-crRNA is not activated. No cleavage of reRNA occurs in the solution, and the reRNA is removed by streptavidin (SA)-coated magnetic beads. Therefore, almost no electrochemical signal can be observed.

The principle of the developed electrochemical biosensor is shown in Figure 1. The strategy involves using guide CRISPR RNA (crRNA) designed to specifically recognize a target RNA, for example, SARS-CoV-2 RNA. After the Cas protein recognizes the target RNA, trans-cleavage activity is activated, resulting in the cleavage of reporter RNA (termed reRNA), which is a 20 nucleotide (nt) ssRNA (poly U) that modified with electrochemically activated biotin on the 5′ end and MB on the 3′ end (termed biotin-ssRNA-MB). After trans-cleavage activity, SA-coated magnetic beads are used to bind the reRNA through the biotin moiety and remove any uncleaved reRNA and biotin-oligonucleotide in the reaction solution by magnetic separation, leaving the MB-oligonucleotide in the solution. Subsequently, an electrochemical workstation and a three-electrode commercial screen-printed carbon electrode were applied to detect the MB electrochemical signal in the mixture, leading to the ultrasensitive and specific detection of the target RNA.

Using SARS-CoV-2 RNA as the target, the trans-cleavage activity of Cas13a was activated by RT-RAA-amplified and T7 transcribed target nucleic acid, resulting in the cleavage of the reRNA. The reRNA was cleaved into MB-oligonucleotide and biotin-oligonucleotide. The remaining uncleaved reRNA and biotin-oligonucleotide were removed by the SA-coated magnetic beads, leaving the MB-oligonucleotide in solution. At this stage, an electrochemical signal is acquired by SWV, and the redox peak signal associated with the MB concentration is recorded. In turn, in the absence of target SARS-CoV-2 RNA, an MB redox peak signal is not observed. In combination with RT-RAA, this novel electrochemical CRISPR biosensor could be used as a low-cost, easy-to-fabricate, ultrasensitive, and highly specific biosensor for the detection of SARS-CoV-2 as well as other pathogens.

### 3.2. Verification of the Electrochemical CRISPR Biosensor

**Figure 2:**
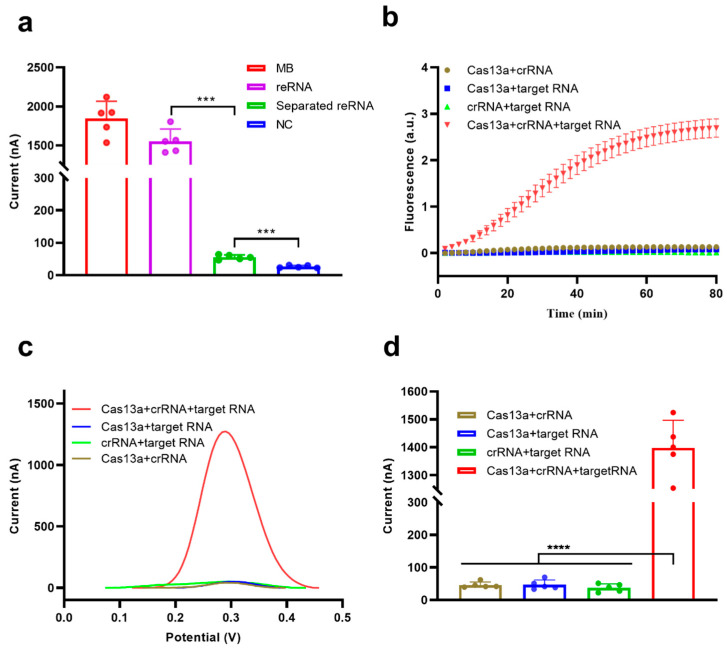
Feasibility analysis of the electrochemical CRISPR biosensor. (**a**) Feasibility analysis of the separation/removal of reRNA using SA-coated magnetic beads. (**b**) Real-time fluorescence analysis of the feasibility of the Cas13a system. (**c**) Example electrochemical signal and (**d**) Square wave voltammetry (SWV) analysis of the feasibility of the Cas13a system for reRNA cleavage. Values represent means ± Standard Deviation (SD), where *n* = 5 replicates. An unpaired two-tailed Welch’s *t*-test was used to analyze statistical significance (*** *p* < 0.001, **** *p* < 0.0001).

To validate the feasibility of this electrochemical CRISPR biosensor, the SA-coated magnetic beads were used to enrich the amount of trans-cleavage-associated electrochemically active molecule (MB in this study). This was achieved through the addition of abundant reRNA, followed by the separation of uncleaved reRNA. The reRNA separation ability of the SA-coated magnetic beads was evaluated by measuring the SWV electrochemical signals of MB and reRNA in solution and the reRNA solution after treatment with SA-coated magnetic beads (all in a NaCl solution) and compared with the signals obtained using an NC sample (NaCl solution only) (Figure 2a). A significant difference in electrochemical signal was observed between the MB and NC samples (*p* < 0.001), indicating the participation of MB in the redox reaction on the surface of the screen-printed carbon electrode. The reRNA sample also exhibited a strong electrochemical signal response, but slightly less than the pure MB sample. This was mainly due to a reduction in diffusion velocity with increasing molecule size because MB is much smaller than MB-ssRNA-biotin and therefore diffuses faster [29]. Almost no electrochemical signal response was observed for the reRNA sample after separation with the magnetic beads, demonstrating that intact reRNA could be efficiently removed from the solution by the SA-coated magnetic beads.

To further examine the feasibility of the biosensor for nucleic acid detection, we selected the N gene of SARS-CoV-2 as the target RNA. Cas13a trans-cleavage activity was activated in the presence of target RNA, and the cleavage of reRNA could be monitored by real-time fluorescence monitoring (Figure 2b) and electrochemical measurements (Figure 2c,d) by reading a significant fluorescence signal or electrochemical peak. The fluorescence and electrochemical signals barely changed in the absence of Cas13a, crRNA, or 10,000 pM target RNA (Figure 2b–d). The amplitude of the electrochemical peak reflected the degree of reRNA cleavage. The average electrochemical peak current of the Cas13a + crRNA + target RNA group reached 1398.0 nA after CRISPR/Cas13a trans-cleavage activity, which is 30-fold higher than the average peak current of the Cas13a + crRNA group (46.1 nA), indicating excellent feasibility for this detection platform (Figure 2d).

### 3.3. Optimization of the Reaction Conditions

In this electrochemical CRISPR biosensor, the separation and removal of extra, uncleaved reRNA are key to distinguishing positive and negative results. Therefore, the ability of SA-coated magnetic beads to bind the reRNA in the solution and then remove it was evaluated (1.7 mol/L NaCl served as the electrolyte) (Figure S1a). The SA-coated magnetic beads efficiently removed the reRNA in solution as indicated by a decrease in the electrochemical signal; after 1 min of incubation, the electrochemical signal was barely detected. Therefore, an incubation time of 1 min with SA-coated magnetic beads was used in this study.

Divalent Mg ions not only play an important role in DNA polymerase catalysis during RT-RAA isothermal amplification [30], but they also affect the cleavage activity of Cas13a [26]. We analyzed a 0–30 mM Mg^2+^ concentration gradient for verification and found that 25 mM Mg^2+^ yielded the best detection performance (Figure S1b). This result is close to the 20 mM Mg^2+^ suggested in one previous Cas12a study [12]. Therefore, we selected 25 mM Mg^2+^ for further investigation in this study. Different concentrations of Cas13a protein were also evaluated, revealing that the electrochemical current increased with an increase in Cas13a protein concentration. For cost considerations, we selected the concentration of 75 mM Cas13a protein for use with the electrochemical CRISPR biosensor (Figure S1c).

### 3.4. Performance Evaluation

#### 3.4.1. Electrochemical CRISPR Biosensor without Target RNA Amplification

**Figure 3:**
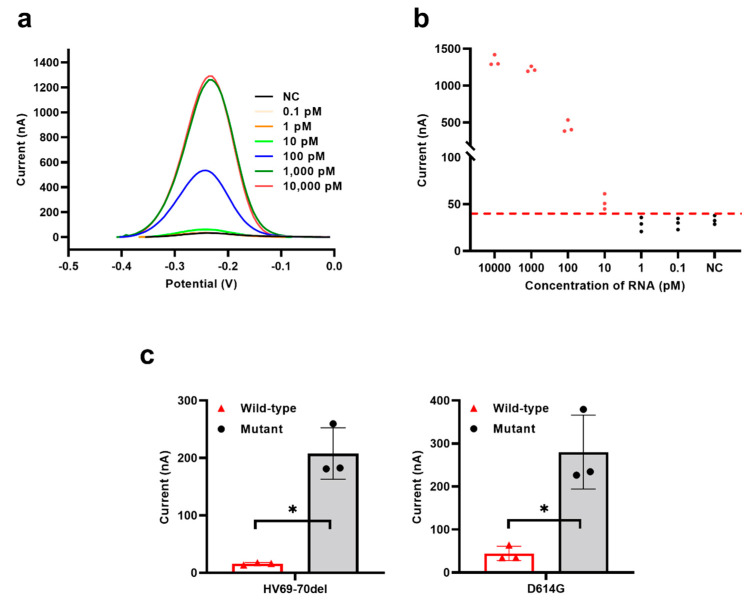
Performance evaluation of the electrochemical CRISPR biosensor without target RNA amplification. (**a**) SWV signals to various concentrations of target RNA from 0.1 pM to 10,000 pM. (**b**) Sensitivity analysis, corresponding to (**a**). (**c**) Specificity analysis between wild-type strain between two different SARS-CoV-2 variant sites strain(HV69-70del and D614G). The red horizontal dashed line (**b**) indicates the cutoff current value defined by the average value of the negative control without target RNA (NC) plus three times the SD. The red-filled dots (**b**) means the current value was larger than the cutoff current value, while the black-filled dots (**b**) means the current value was smaller than the cutoff current. Values represent means ± SD, where *n* = 3 replicates. An unpaired two-tailed Welch’s *t*-test was used to analyze the statistical significance.

Under optimized experimental conditions, we investigated the performance of the electrochemical CRISPR biosensor without target RNA amplification. The electrochemical current cutoff value was calculated to be 44.4 nA (average background value plus three times the standard deviation). Samples containing 10,000, 1000, 100, and 10 pM RNA had a higher electrochemical current than the cutoff value (Figure 3a,b), indicating that the target RNA in these samples was successfully detected. The average electrochemical current values between 10,000 pM and 1000 pM groups were almost equal; this result may be because all of the reRNA was successfully cleaved, and the highest electrochemical current value had been reached in both groups. Compared with other electrochemical CRISPR biosensors in detecting unamplified target RNA, the biosensor developed here showed an improved detection limit (down to 10 pM target RNA) [4,20]. The developed biosensor benefits from both an efficient reaction in a homogeneous solution and a significant reduction in the background signal by the removal of extra reRNA. More specifically, in our system, compared to heterogeneous electrochemical methods, analyte recognition and signal amplification transduction are performed in a homogeneous solution, which avoids steric hindrance of Cas13a interfacial cleavage [31]. In addition, our experimental procedure is simplified, effectively improving measurement reliability and repeatability of the biosensors. When SA-coated magnetic beads were used to remove the remaining reRNA, the background signal was greatly reduced, and the signal-to-noise ratio was improved, which was beneficial to signal output.

Furthermore, we verified the assay’s specificity by designing and testing two different SARS-CoV-2 variant sites (HV69-70del and D614G). Positive results were obtained only when mutant or wild-type SARS-CoV-2 sequences matched their respective crRNAs, and no obvious cross-reactivity was observed. The HV69-70del SARS-CoV-2 mutant strain was missing six bases compared to the wild-type strain (Figure S2a), and the D614G SARS-CoV-2 mutant strain had a single base exchange (Figure S2b). As indicated in Figure 3c, mutant-type SARS-CoV-2 with the corresponding crRNA induced specific electrochemical responses. When the crRNA of the mutant-type SARS-CoV-2 strains bound to the wild-type strain sequence, the wild-type samples showed a lower electrochemical signal. These results showed that the NucID strain had a single base exchange or missing six bases that could be distinguished by this biosensor, which demonstrates that the biosensor has high specificity. Additionally, owing to the programmability of crRNA, the electrochemical CRISPR biosensor allows for the development of a universal biosensing platform for any target RNA by easily changing the sequence of the crRNA guide region [32].

#### 3.4.2. Electrochemical CRISPR Biosensor with RT-RAA Amplification

The RT-RAA is an isothermal nucleic acid detection method that is both fast and robust; it is based on an enzyme reaction that has been successfully applied in the highly sensitive detection of pathogens [3,33,34]. Together with the signal amplification of Cas13a trans-cleavage activity [35], the RT-RAA-based CRISPR/Cas13a electrochemical biosensor described here exhibited very good sensitivity. When the electrochemical CRISPR biosensor was combined with RT-RAA isothermal amplification, T7 transcription was required for trans-cleavage activity, a time-consuming process. However, for the purpose of POCT, the whole operating time should not be very long. Based on our previous study [27], 30 min was sufficient for T7 transcription and trans-cleavage. In practice, the entire process required no more than 63 min, broken down as follows: RT-RAA isothermal amplification to amplify the target nucleic acid (30 min), T7 transcription and Cas13a cleaving time (30 min), removal of excess reRNA with SA-coated magnetic beans (1 min), and measurement of the electrochemical signal (2 min).

**Figure 4:**
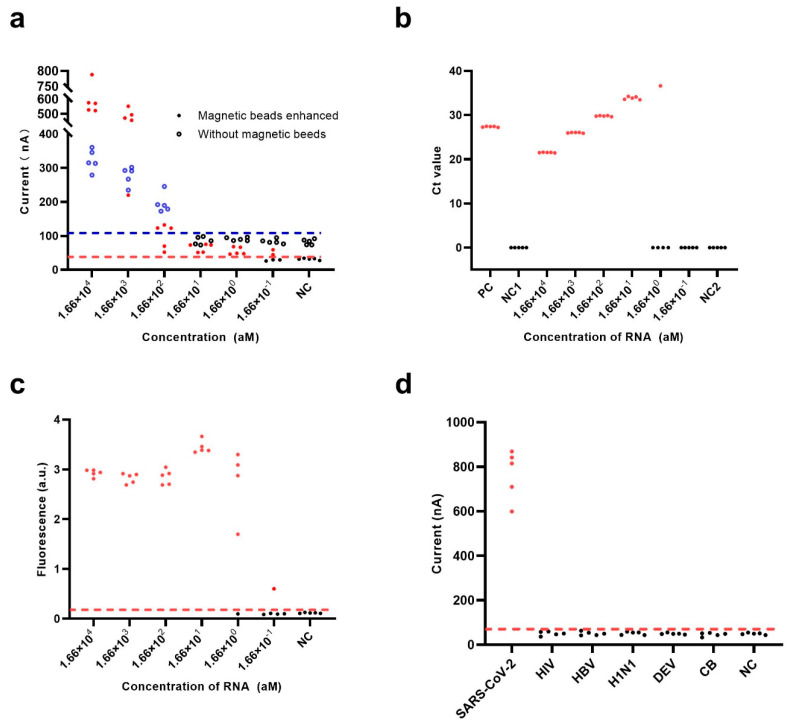
Performance evaluation of the electrochemical CRISPR biosensor with RT-RAA amplification. Sensitivity analysis of (**a**) the electrochemical CRISPR biosensor with (filled dots) or without (hollow dots) SA-coated magnetic beads enhancement, (**b**) the RT-QPCR assay, and (**c**) the fluorescent CRISPR assay were performed with various concentrations of target RNA from 0.166 aM to 1.66 × 10^4^ aM. (**d**) Specificity analysis with 1.66 × 10^4^ aM RNA of SARS-CoV-2, human immunodeficiency virus (HIV), hepatitis B virus (HBV), hemagglutinin 1 neuraminidase 1 (H1N1), dengue virus (DENV), and Coxiella burnetii (CB). The red horizontal dashed line indicates the cutoff current value of the electrochemical CRISPR biosensor with magnetic beads enhanced (**a**,**d**) and the fluorescent CRISPR assay (**c**). The blue horizontal dashed line (**a**) indicates the cutoff current value of the electrochemical CRISPR biosensor without magnetic beads enhanced. The red-filled dots (**a**–**d**) and hollow blue dots (**a**) indicate positive results, and the solid black dots (**a**–**d**) and black hollow dots (**a**) indicate negative results in the electrochemical CRISPR biosensor with magnetic beads enhanced assay, electrochemical CRISPR biosensor without SA-coated magnetic beads enhancement, the RT-QPCR assay and the fluorescent CRISPR assay.

Under optimized experimental conditions and successful nucleic acid amplification (Figure S3), we investigated the performance of the electrochemical CRISPR biosensor both with and without SA-coated magnetic bead enhancement against a range of different concentrations of target RNA. Coronavirus disease 2019 (COVID-19) RNA reference material (high concentration) containing the SARS-CoV-2 N gene sequence was used to compare the ability of the biosensor to detect RNA using a fluorescent CRISPR assay and RT-qPCR. As shown in Figure 4a, the SA-coated magnetic beads demonstrated a remarkable ability to reduce the background noise signal, while the NC group with SA-coated magnetic beads enhancement demonstrated a lower level of background noise signal than the NC group without SA-coated magnetic beads enhancement. Moreover, the electrochemical CRISPR biosensor with SA-coated magnetic bead enhancement could detect down to 1.66 × 10^0^ aM of COVID-19 RNA reference material, while the biosensor without magnetic bead enhancement could only detect down to 1.66 × 10^2^ aM of reference material.

Currently, RT-qPCR is considered the “gold standard” for COVID-19 diagnosis by the United States Center for Disease Control and Prevention (CDC) [36]; thus, the ability of RT-qPCR to detect the same amount of COVID-19 RNA reference material as that detected using the electrochemical CRISPR biosensor was also evaluated. However, RT-qPCR could not detect 1.66 × 10^0^ aM; the detection limit was 1.66 × 10^1^ aM of COVID-19 RNA reference material (Figure 4b), which is consistent with the results released by the CDC [36]. Notably, the detection limit using intact RT-qPCR methods for diagnosis may even be less than 0.83 aM (i.e., 0.5 copies/μL), especially when using a nucleic acid extraction procedure that can concentrate the samples about 5–10 times. However, since the samples in this study were COVID-19 RNA reference material, we did not need to extract the nucleic acid; hence these samples were not concentrated. This explains that the RT-qPCR detection limit of 1.66 × 10^1^ aM for COVID-19 RNA reference material is reasonable. This experimental result also shows that the sensitivity of the electrochemical CRISPR biosensor was better than that of RT-qPCR, and the results were more stable than those using the fluorescent CRISPR method.

As a classic nucleic acid test, the fluorescent CRISPR assay was also used to detect COVID-19 RNA reference material. The results showed that in the 1.66 × 10^0^ aM group, all but one of the five samples were positive, which demonstrates the fluorescent CRISPR assay could only detect down to 1.66 × 10^1^ aM reference material (Figure 4c).

In addition to the flexible programmability of crRNA and the effective signal amplification efficiency of Cas13a, the high selectivity of the Cas13a/crRNA system is another important advantage. It was previously confirmed that the Cas13a/crRNA-based direct nucleic acid detection systems have single-base resolution [37]. In this study, the specificity of the electrochemical CRISPR biosensor with RT-RAA amplification was investigated using six different pathogens: SARS-CoV-2, human immunodeficiency virus (HIV), hepatitis B virus (HBV), hemagglutinin 1 neuraminidase 1 (H1N1), dengue virus (DENV), and Coxiella burnetii (CB). The target RNA extracted from SARS-CoV-2 exhibited a significant positive signal, whereas negative signals were obtained for the other pathogens, comparable to the blank control. These results indicate the superior specificity of the electrochemical CRISPR biosensor with RT-RAA amplification (Figure 4d).

### 3.5. Analysis of Clinical Samples

**Figure 5:**
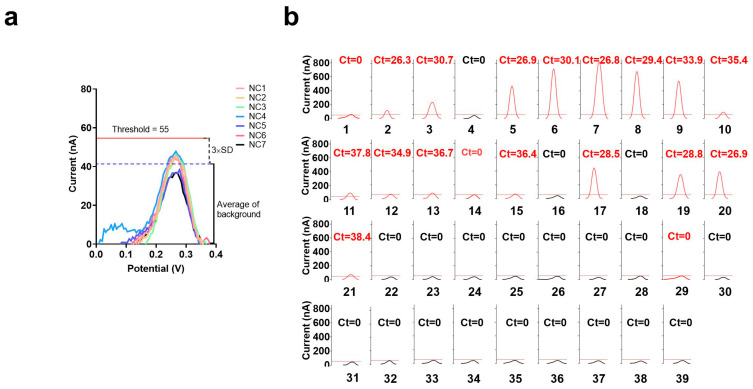
Analysis of clinical samples. (**a**) Electrochemical CRISPR biosensor with RT-RAA amplification test results of seven clinical samples from healthy individuals, NC1 through NC7, are the clinical samples from healthy individuals. (**b**) Thirty-nine suspected positive clinical samples were also tested using the electrochemical CRISPR biosensor with RT-RAA amplification and real-time fluorescence RT-qPCR (Ct value on each small panel, red and black Ct value represent the sample was judged as positive or negative by the electrochemical CRISPR biosensor with RT-RAA amplification). Sample numbers 1–39 successively represent 39 clinical sample numbers. The horizontal dashed line indicates the threshold (value is 55nA). The curve is the SWV signal of the electrochemical CRISPR biosensor test results. The red curve indicates that the maximum current value exceeds the threshold and is judged as positive. Conversely, the black curve indicates that its maximum current value is less than the threshold value, and the result is judged negative.

To achieve a better clinical diagnosis, we introduced the concept of a cutoff value, which was set to the background (based on the average current value of seven health clinic samples) plus three times the SD. The experimental results showed that the cutoff value for positivity was 55 nA (Figure 5a). When the current value of the electrochemical CRISPR biosensor with RT-RAA amplification was >55 nA, it was considered a positive result, and a current value of ≤55 nA was considered a negative result. A total of 39 clinical samples from the Beijing Institute of Microbiology and Epidemiology were analyzed using the electrochemical CRISPR biosensor and RT-qPCR (Figure 5b).

Samples 2, 3, 5–13, 15, 17, and 19–21 were detected both positive by electrochemical CRISPR biosensor with RT-RAA amplification and real-time fluorescence RT-qPCR. Samples 4, 22–28, and 30–39 were detected as negative using an electrochemical CRISPR biosensor with RT-RAA amplification and real-time fluorescence RT-qPCR. However, samples 1, 14, and 29 were detected positive only by electrochemical CRISPR biosensor with RT-RAA amplification. So we can get the detailed numbers in Table 1. The electrochemical CRISPR biosensor yielded 19 positive samples and 20 negative samples, whereas real-time fluorescent RT-qPCR yielded 16 positive samples and 23 negative samples. The coincidence rate was 92.3% (Table 1). A total of three samples tested negative using RT-qPCR but positive using the electrochemical CRISPR biosensor. Further, these same three samples were additionally tested using Droplet Digital PCR, and all three samples tested positive (Table 2), which was consistent with the electrochemical CRISPR biosensor results.

## 4. Conclusions

In this study, we developed an immunocapture magnetic bead-enhanced biosensor that uses both isothermal amplification and CRISPR-based detection to test for SARS-CoV-2. This biosensor enables rapid, ultrasensitive (down to ~1.66 × 10^0^ aM), and highly specific nucleic acid detection. We attribute the rapid detection speed and ultrahigh sensitivity of the biosensor to the following: (i) the immunocapture magnetic beads reduce the background noise signal while allowing an abundant concentration of cleaved reRNA in the positive group, (ii) the system enables CRISPR-Cas13a cleavage within the homogeneous solution rather than at the electrode/heterogeneous solution interface, and (iii) RAA amplification and CRISPR-Cas13a-based activity can be combined. Additionally, the biosensor uses a commercial screen-printed carbon electrode, which does not require pre-treatment or molecular immobilization, making the biosensor easy to fabricate and operate while saving time overall. Based on the results of this study, the immunocapture magnetic bead-enhanced electrochemical biosensor for ultrasensitive SARS-CoV-2 detection based on CRISPR-Cas13a technology represents a promising electrochemical nucleic acid detection strategy for clinical application at the point of care.

## Figures and Tables

**Table 1:** Clinical validation (nucleic acid samples) of the electrochemical CRISPR biosensor for detecting SARS-CoV-2 compared with RT-qPCR results.

		RT-qPCR		Coincidence Rate
		Positive	Negative	
Electrochemical CRISPR biosensor	Positive	16	3	92.3%
	Negative	0	20	
	Total	16	23	

**Table 2:** Droplet Digital PCR results for three suspected positive cases of SARS-CoV-2 infection as detected using the electrochemical CRISPR biosensor.

	#1	#14	#29
N gene copy number	0.087 copies/μL	0.85 copies/μL	0.15 copies/μL
Result	Positive	Positive	Positive

## Data Availability

All data supporting the findings of this study are available from the corresponding authors upon reasonable request (email:hanyaohyhy@163.com).

## Supplementary Materials

The following supporting information can be downloaded at: https://www.mdpi.com/article/10.3390/bios13060597/s1, Figure S1: Optimization of the electrochemical CRISPR biosensor; Figure S2: The sequence of SARS-CoV-2 variant sites (HV69-70del and D614G) and the crRNA; Figure S3: Amplification of the N gene of SARS-CoV-2 by RT-RAA; Table S1: Sequences involved in this study; Table S2: The detailed results of several methods testing on clinical samples.
