# Supplementary material for: Imunocapture Magnetic Beads Enhanced and Ultrasensitive CRISPR-Cas13a-Assisted Electrochemical Biosensor for Rapid Detection of SARS-CoV-2
Source: Biosensors (Basel). 2023 May 31;13(6):597. doi: 10.3390/bios13060597 (PMC10296353; doi:10.3390/bios13060597)
Supplement: Supplementary file 1 [file biosensors-13-00597-s001.zip › biosensors-2406627-supplementary.pdf]

Supplementary Material

Imunocapture Magnetic Beads Enhanced and Ultrasensitive CRISPR-Cas13a-Assisted Electrochemical Biosensor for Rapid Detection of SARS-CoV-2

Yao Han [1,†], Fan Li [1,†], Lan Yang [1,†], Xudong Guo [2], Xue Dong [1], Mengwei Niu [1], Yaxuan Jiang [3], Lin Li [1,*], Hao Li [1], and Yansong Sun [1,*]

[1] State Key Laboratory of Pathogen and Biosecurity, Beijing Institute of Microbiology and Epidemiology, Beijing 100071, China; hanyaohyhy@163.com (Y.H.); lifan910129@163.com (F.L.); poplarorchid@163.com (L.Y.); dongxue941003@163.com (X.D.); n17865312968@outlook.com (M.N.); lihao1@bmi.ac.cn (H.L.)

[2] Chinese PLA Center for Disease Control and Prevention, Beijing 102206, China; 18601962452@163.com

[3] College of Public Health, Zhengzhou University, Zhengzhou City 450001, China; 18895692187@163.com

* Correspondence: lilin1@bmi.ac.cn (L.L.); sunys6443@126.com (Y.S.)

† These authors contributed equally to this work.

Table S1. Sequences involved in this study

Name	Sequence (5'-3')
Target sequence (SARS-CoV-2)	AACUAAUCAGACAAGGAACUGAUUACAAACAUUGGCCGCAAAUUGCACAAUUUGCCCCCAGCGCUUCAGCGUUCUUCGGAAUGUCGCGCAUUGGCAUGGAAGUCACACCUUCGGGAACGUGGUUGACCUACACAGGUGCCAUCAAAUUGGAUGACAAAGAUCCAAAUUUCAAAGAUCAAGUCAUUUUGCUGAAUAAG
RT-RAA Forward	aattctaatacgactcactatagggAATTGCACAATTTGCCCCCAGCGCTTCAG
RT-RAA Reverse	CTTGATCTTTGAAATTTGGATCTTTGTCAT
RNA-FQ reporter	/56-FAM/UUUUUUUUUUUUUUUUUUUU/3BHQ1/
crRNA	GGGAUUUAGACUACCCCAAAAACGAAGGGGACUAAAACUCCAAUUUGAUGGCACCUGUGUAGGUCA
Biotin-RNA-MB reporter	Biotin/UUUUUUUUUUUUUUUUUUUU/MB

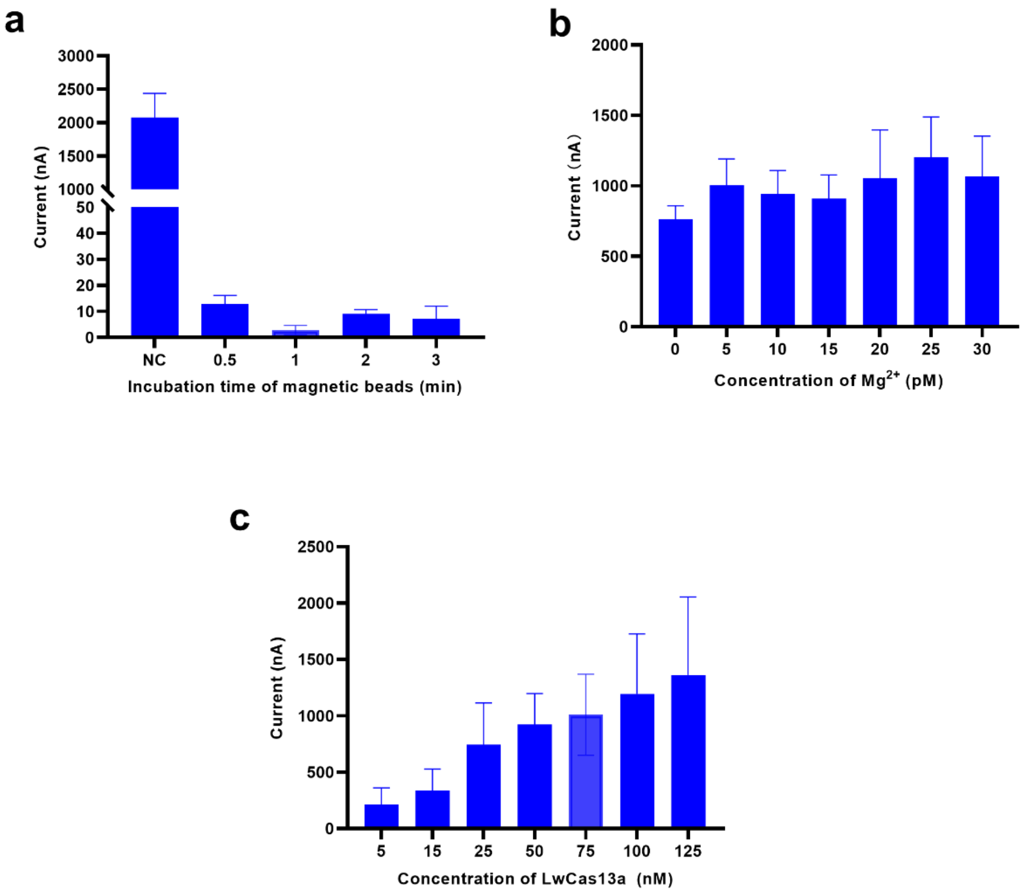

Figure S1. Optimization of the electrochemical CRISPR biosensor. (**a**) Incubation time of magnetic beads with reRNA, concentration of Mg ion (**b**) and Cas13a protein (**c**) used in the mixture were evaluated. NC is the negative control without separation with streptavidin magnetic beads. Error bars represent mean ± SD, where n =3 or 5 replicates.

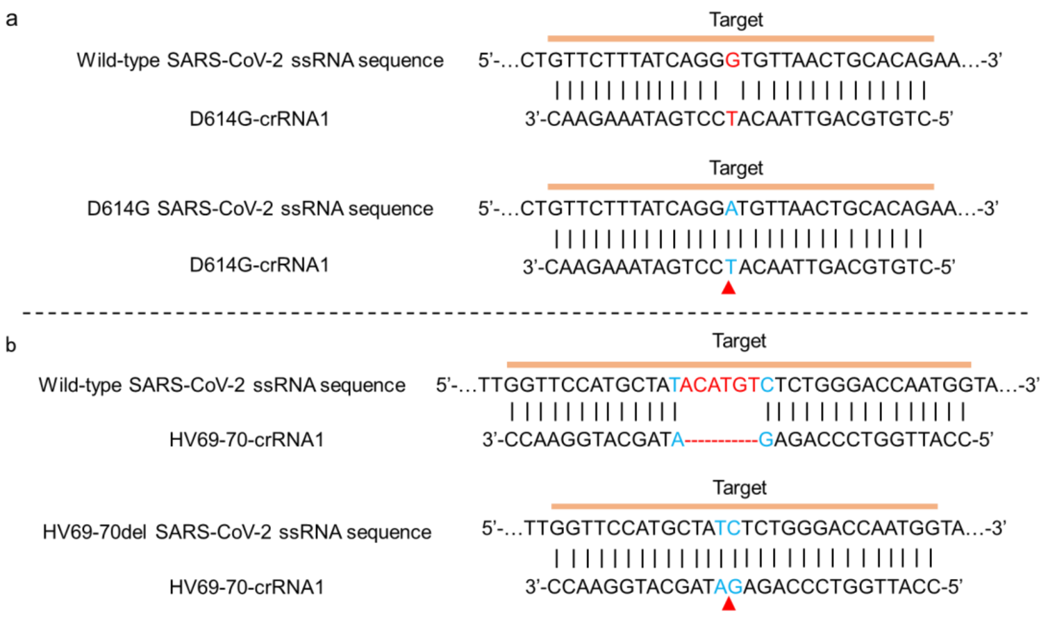

Figure S2. The sequence of SARS-CoV-2 variant sites (HV69-70del and D614G) and the crRNA.

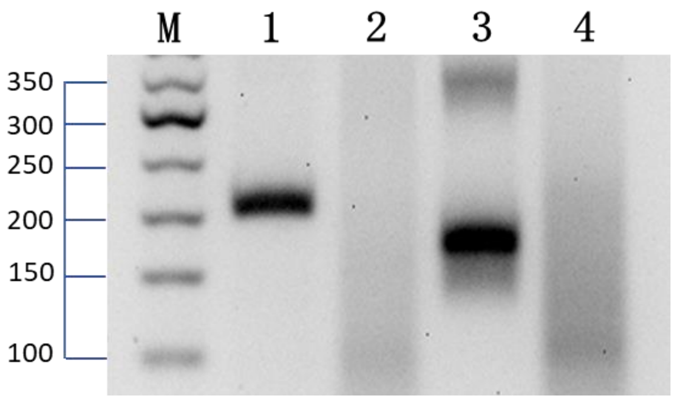

Figure S3 Amplification of the N gene of SARS-CoV-2 by RT-RAA. Lanes 1 and 2 represent negative and positive control in the RT-RAA amplification kit, respectively; Lanes 3 and 4 correspond to RT-RAA amplification of the N gene and its negative control, respectively; Lane M is the DL500 DNA marker.

Table S2. The detailed results of several methods testing on clinical samples.

NO.	Ct value	Current（nA）	NO.	Ct value	Current（nA）	NO.	Ct value	Current（nA）
**1**	0	57.5	**19**	28.8	349.4	**37**	0	37.6
**2**	26.33	118.6	**20**	26.92	388.6	**38**	0	35.0
**3**	30.69	235.7	**21**	38.36	71.5	**39**	0	40.9
**4**	0	42.8	**22**	0	39.5	**NC 1**	0	45.4
**5**	26.92	471.0	**23**	0	42.8	**NC 2**	0	44.3
**6**	30.14	718.5	**24**	0	41.4	**NC 3**	0	46.5
**7**	26.75	821.2	**25**	0	49.1	**NC 4**	0	47.9
**8**	29.42	680.2	**26**	0	50.4	**NC 5**	0	38.5
**9**	33.85	543.1	**27**	0	34.7	**NC 6**	0	45
**10**	35.4	93.0	**28**	0	53.9	**NC 7**	0	36.9
**11**	37.78	96.6	**29**	0	56.5			
**12**	34.89	64.6	**30**	0	33.3			
**13**	36.67	81.3	**31**	0	45.2			
**14**	0	57.1	**32**	0	51.8			
**15**	36.44	67.6	**33**	0	44.6			
**16**	0	43.2	**34**	0	40.3			
**17**	28.54	443.0	**35**	0	43.7			
**18**	0	37.1	**36**	0	41.5			
